# Photobiomodulation Therapy in Oral Mucositis and Potentially Malignant Oral Lesions: A Therapy Towards the Future

**DOI:** 10.3390/cancers12071949

**Published:** 2020-07-18

**Authors:** Reem Hanna, Snehal Dalvi, Stefano Benedicenti, Andrea Amaroli, Tudor Sălăgean, Ioana Delia Pop, Doina Todea, Ioana Roxana Bordea

**Affiliations:** 1 Department of Surgical Sciences and Integrated Diagnostics, Laser Therapy Centre, University of Genoa, Viale Benedetto XV,6, 16132 Genoa, Italy; drsnehaldeotale@gmail.com (S.D.); stefano.benedicenti@unige.it (S.B.); 2 Department of Oral Surgery, Dental Institute, King’s College Hospital NHS Foundation Trust, London SE5 9RS, UK; 3 Department of Periodontology, Swargiya Dadasaheb Kalmegh Smruti Dental College and Hospital, Nagpur 441110, India; 4 Department of Orthopaedic Dentistry, First Moscow State Medical University (Sechenov University), Trubetzkaya Street, 8, Bldg. 2, 119146 Moscow, Russia; andrea.amaroli71@gmail.com; 5 Department of Land Measurements and Exact Sciences, University of Agricultural Sciences and Veterinary Medicine Cluj-Napoca, 400372 Cluj-Napoca, Romania; tudor.salagean@usamvcluj.ro (T.S.); popioana@usamvcluj.ro (I.D.P.); 6 Department of Pulmonology, “Iuliu Hațieganu” University of Medicine and Pharmacy Cluj-Napoca, 400332 Cluj-Napoca, Romania; doina_adina@yahoo.com; 7 Department of Oral Rehabilitation, “Iuliu Hațieganu” University of Medicine and Pharmacy Cluj-Napoca, 400012 Cluj-Napoca, Romania; roxana.bordea@ymail.com

**Keywords:** head and neck cancer, radiotherapy, low-level laser therapy, oral mucositis, oral lichen planus, oral leukoplakia, phototherapy toxicity, photobiomodulation, PBMT, oral squamous cell carcinoma

## Abstract

Photobiomodulation therapy (PBMT) is an effective treatment modality, which has the significant advantage of enhancing a patient’s quality of life (QoL) by minimising the side effects of oral cancer treatments, as well as assisting in the management of potentially cancerous lesions. It is important to note that the major evidence-based documentation neither considers, nor tackles, the issues related to the impact of PBMT on tumour progression and on the downregulation of cellular proliferation improvement, by identifying the dose- and time-dependency. Moreover, little is known about the risk of this therapy and its safety when it is applied to the tumour, or the impact on the factor of QoL. The review aimed to address the benefits and limitations of PBMT in premalignant oral lesions, as well as the conflicting evidence concerning the relationship between tumour cell proliferation and the applied dose of photonic energy (fluence) in treating oral mucositis induced by head and neck cancer (H&N) treatments. The objective was to appraise the current concept of PBMT safety in the long-term, along with its latent impact on tumour reaction. This review highlighted the gap in the literature and broaden the knowledge of the current clinical evidence-based practice, and effectiveness, of PBMT in H&N oncology patients. As a result, the authors concluded that PBMT is a promising treatment modality. However, due to the heterogeneity of our data, it needs to undergo further testing in well-designed, long-term and randomised controlled trial studies, to evaluate it with diligent and impartial outcomes, and ensure laser irradiation’s safety at the tumour site.

## 1. Introduction

### 1.1. Photobiomodulation Mechanism of Action

**Figure 1:**
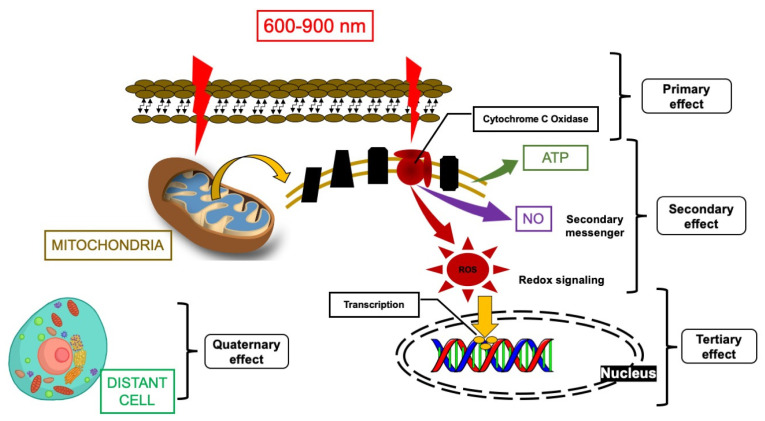
Modified schematic description of the PBMT’s effects on stressed tissue, highlighted in 4 stages: primary effects (photonic energy absorption by cytochrome C Oxidase (CCO)), secondary effects (mitochondrion of ATP, NO, and ROS), tertiary effects (downstream of intracellular responses (Gene transcription and cellular signaling)), and quaternary effect (indirect/distant effects) [4]. Abbreviations: PBM, photobiomodulation; ROS, reactive oxygen species; NO, nitric oxide; ATP, adenosine triphosphate.

Photobiomodulation (PBM) is a manipulation of the molecular and cellular activities of a tissue irradiated by the photonic energy of a non-ionising light source (light-biological tissue interactions), which generates a non-thermal therapeutic effect. The effect of photobiomodulation therapy (PBMT) on stressed tissue can be described in four phases (Figure 1). The primary effect was addressed first by Karu et al., 2010, and is related to the peak absorption of the red (600–700 nm) and near-infrared (NIR (760–900)) photonic energy by cytochrome C oxidase (CCO), which subsequently triggers a series of downstream effects [1,2]. Therefore, the therapeutic window for PBM ranges from 600 to 900 nm (usually at a low power in the range of 1–500 mW) [3,4]. The secondary effect refers to the changes in the adenosine triphosphate (ATP), nitric oxide (NO) and reactive oxygen species (ROS), and whether they follow the light photonic energy’s absorption by CCO. These effects are dependent on dose and redox states [5]. The tertiary effect is related to the downstream effects [4]. These effects are context- and cell-type-specific, and also act in a direct or indirect way. These biochemical events affect the cell membrane and nucleus, which control gene transcription and subsequently cell proliferation, migration, apoptosis and inflammation [6]. The quaternary effects (distant, systemic effects) of PBMT are associated with the tissues that have not absorbed the photonic energy, yet can still be affected indirectly by the secretions of the cells that have absorbed the laser light [4]. Hence, the main clinical questions, which can be formulated for optimising the treatment of patients with suspected oral lesion, are as follows:

(1) How can PBMT be improved to identify the dose- and time-dependent conditions, and the laser therapeutic protocol, for down-regulating the cellular proliferation of suspected lesions and achieving optimal outcomes?
(2) What is the effect of PBMT on tumour growth?
(3) How can PBMT be a useful modality in enhancing a patient’s clinical outcome and improving their quality of life (QoL)?
(4) How can PBMT be useful as a monotherapy, or as an adjunctive modality, in treating potential malignant lesions?
(5) What are the benefits in, and necessary precautions for, utilising PBMT in potentially malignant oral lesions?

### 1.2. PBM-Influencing Factors for Treatment Optimisation

The optimal therapeutic treatments using PBM can be influenced by tissue properties [7]. In this context, it is important to note that each wavelength, at a certain fluence (energy density), has a different penetration depth with regards to reaching the target tissue, and taking into account the tissue components, ultimately, there is a percentage of dispersion of the energy [8]. Therefore, it is important to consider the nature of the lesion in terms of consistency, composition and location (deep or superficial). Moreover, the influencing factors that have a significant impact on the optimisation of the PBM therapeutic outcomes can be considered both in terms of biological and clinical considerations (Table 1) [9]. Hanna et al., 2019, conducted an in-vitro study to show that the therapeutic power output (measured with a power meter), dose (fluence) and the beam profile play crucial roles in achieving the optimal therapeutic outcome [10]. The clinical treatment rationale can be optimised by attention, in order to induce a biological response.

**Figure 2:**
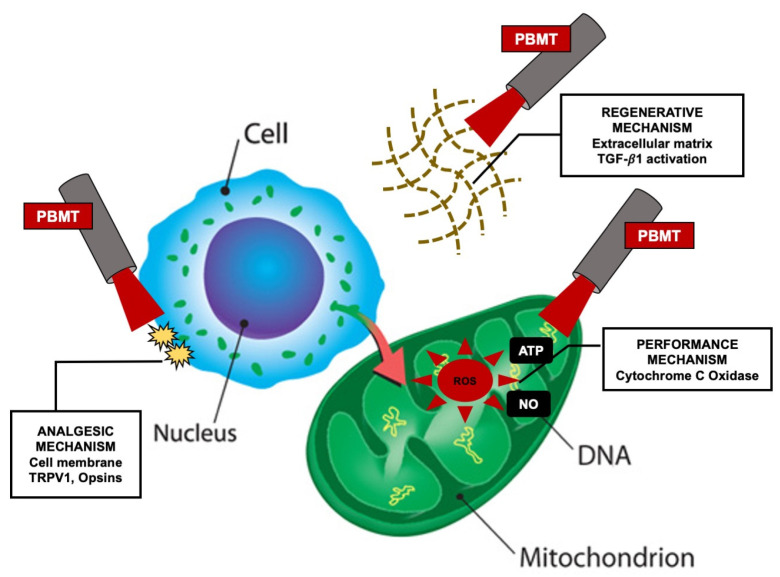
Modified schematic description of the three discrete PBM mechanisms of action [11] Abbreviations: TRPV1, transient receptor potential cation channel subfamily V member1; TGF-β, transforming growth factor beta; PBM, photobiomodulation; ROS, reactive oxygen species; NO, nitric oxide; ATP, adenosine triphosphate.

Arany et al., 2014, suggested that there are three correlated PBM mechanisms at the molecular level that enable specific biological responses [11]. While each mechanism emerges to govern a defined therapeutic applications (performance, analgesics, regenerative), there is growing evidence that there is a significant crosstalk among them. These scenarios occur within the cell, on the cell membrane and in the extracellular matrix [11,12] (Figure 2).

It is also important to note that the fundamental mechanisms of the biochemistry phenomena of PBMT responses remain incompletely developed. Studies have proven that PBM has a significant impact on cellular and molecular activities, and the mode of action differs among various applications [10,11,12,13]. 

### 1.3. PBMT and Cancer 

There is significant commentary and debate surrounding the available evidence related to the possibility of the potential long-term risks of PBMT in cancer cell mutation and amplification, or escalated relapse rates [14]. Whether PBM irradiation, directly in line with zones anatomically associated with the tumour, has a negative or a positive impact on the behaviour of the tumour cells, and/or on the treatment response, is presently unclear. Hence, in this context, caution is necessary, due to the currently proposed biological mechanisms of action of PBM, the conflicting in vitro data concerning PBMT-induced tumour mutation, as well as the limited nature of the long-term follow-ups of the clinical evidence-based data in order to confirm the safety of PBMT. Ultimately, in oral mucositis (OM) treatment, these dynamics need to be considered, in order to evaluate the efficacy of this therapy. The review of Sonis et al., 2016, and the study of Antunes et al., 2017 [14,15], showed that the progression-free survival of head and neck (H&N) oncology patients treated with radiotherapy and chemotherapy (RT-CT) increased due to the PBMT [16]. Nevertheless, in this context, until extensive and reliable long-term safety data are available, PBMT should be used with caution when the irradiation beam is in the direction of the tumour zone. 

These questions need to be addressed to enable clinicians to choose the optimal targeted therapy, with a therapeutic dose, for a suitable patient or an appropriate lesion. In this review, the authors focus on responding to these questions in the light of the recently published evidence. Moreover, this review aims to broaden the knowledge regarding the current evidence-based clinical practice and effectiveness of PBMT in H&N oncology patients. The aim was to appraise the current concept of PBMT’s efficacy, its long-term safety, and its potential impact on tumour response. 

## 2. PBMT, Dose-Dependency and Its Correlation with Proliferation Rate in Head and Neck Squamous Cell Carcinoma (HNSCC)

### Data Extracted from In Vitro Molecular and In Vivo Animal Studies

Several animal in vivo studies [17,18] have shown that PBMT can promote collagen synthesis, resulting in wound-healing and a decrease in the levels of cyclooxygenase-2 (COX-2) and neutrophil infiltration into the OM wound. Based on this, Silva et al.’s 2014 study [19] investigated PBM’s effects on inflammatory mediators. It concluded that this therapy enhances interleukin-10 (IL-10) levels. The latter has an anti-inflammatory cytokine effect, thereby reducing the injury triggered by the neutrophils and macrophages. Moreover, this study showed that IL-6 plays an essential role in OM treatment. 

OM is one of the most severe complications for HNSCC patients is the high dose of ionising irradiation, as the primary tumour is present in the field of the PBM’s irradiation during the treatment of oropharyngeal mucositis (ORM) [20]. This could prove to be problematic, irradiating the cancerous cells and subjecting them to stimulatory activities [19,20,21,22]. On this note, the precise cellular and molecular mechanisms by which PBMT induces cell proliferation are, currently, still unproven [23]. The study of Bamps et al., 2018 [24], showed that PBMT, used dose-dependently, increases HNSCC cell proliferation, with no impact on the normal epithelial tonsil cells noted. González-Arriagada et al., 2018 study [25] evaluated the effects of 830-nm diode laser irradiation on SCC154, SQD9 and SCC61 cell lines, and human tonsil epithelial cells, at a power output 150 mW and with various fluences (energy density), including 0, 1 and 2 J/cm^2^. It showed that PBMT at fluence of 1 J/cm^2^ exerts a latent effect to elevate the pAKT, pERK and Ki67 protein expression levels, and cell proliferation levels [23,25], whereas at a fluence of 2 J/cm^2^, no significant increase was noted. This coincides with the study result of Robijns et al., 2017, reporting that PBMT can exhibit a stimulatory response on PI3K and MAPK/ERK [23]. 

Interestingly, the in vitro study of Schalch et al., 2019 [26], aimed to investigate the effect of utilising PBM protocol for OM, with regards to modulating the osteoclastogenic activities of SCC9. The latter is a cell line derived from human lingual SCC. It concluded that SCC9 irradiation with PBM at 780 nm, at a power output 70 mW and a fluence of 4 J/cm², proved to be the safest dose, which led to a decrease in the cell viability, inducing apoptosis and a reduction in tumour metastasis. Nevertheless, there is still some controversy and some concerns regarding the efficacy of PBMT and its impact on inadvertently irradiated potential residual tumour cells. On this note, these studies propose that PBMT can be utilised with a degree of caution when ORM is treated in this cohort. Several in vitro studies have noted that the visible light (red light) of the electromagnetic spectrum, ranging from 622 to 780 nm, is identified as having the ability to mutate and stimulate cell division at a certain fluence [27,28,29], raising some concerns regarding the use of PBMT in zones where tumours have previously been treated [30]. In this context, the study of Henriques et al., 2014 [31], reported a greater proliferation of neoplastic cell lines after irradiation with PBM. The study of Khan et al., 2015, on the other hand, has shown that PBMT did not have any impact on gene toxicity or the mutation process, despite the cytotoxicity related to oxidative stress [32]. This was also demonstrated by the study of Myakishev-Rempel et al., 2012 [33], wherein an ultraviolet-induced skin cancer animal model was utilised. This study investigated whether PBMT can promote tumour growth. In this context, the results reported no quantifiable effects when a 670-nm light emitting diode (LED), employed at a fluence of 5 J/cm^2^, was used twice a day. The experiments suggested the latter protocol is safe even when the malignant lesions were in the path of the irradiation beam [33]. Nonetheless, the study of de C. Monteiro et al., 2012 [34], reported that when a 660-nm laser light irradiated an SCC in a hamster’s cheek pouch, at 56 J/cm^2^ fluence with a 3-mm spot size, a significant increase in the severity of tumour cells was observed, on histological evaluation. On the other hand, a recent clinical study by Guedes et al., 2018 [35], showed that PBMT, at a high laser energy (1.0 J versus 0.25 J), exhibited a small impact on symptom improvement when it was utilised as an OM preventive therapy, without a significant increase in the risk of recurrence in patients undergoing RT for cancer [35]. In addition, there was no correlation between the frequency of recurrences and the energy of the laser irradiation. Regarding this, caution needs to be exercised due to the limited follow-up period. Nonetheless, it is the first step in providing clear evidence for the safe use of PBMT, as a preventive modality of OM-induced by RT. In this context, a better prognosis (progression-free survival) for HNSCC patients, is observed when OM is well-controlled with PBMT [15]. 

Interestingly, PBM therapies (Laser and LED light sources) have emerged as an approach to inhibiting possible cell transformation. Takemoto et al., 2019 [36], aimed to evaluate the effect of high energy density LED-based PBMT in inhibiting an in vitro model (containing stromal fibroblasts and cancerous cells) of squamous cell carcinoma in-situ (CIS). The results showed an inhibition of CIS colony expansion and a reduction in the number of clusters after 72 h of irradiation, at a fluence of 36 J/cm^2^. This recent in vitro study [36] concluded that high-dosage, LED-based PBMT inhibited the progression of the cancerous cells without disturbing the adjacent stromal fibroblasts. This could be a stepping-stone in translational research, which requires further corroboration.

Evidently, there is conflicting evidence regarding the safety of PBMT when the tumour is present in the direct line of beam irradiation. Hence, it is evident that the dose of the photonic energy of the laser/LED-mediated PBM, and time, are fundamental in determining the tumour cells’ proliferations and divisions. On this note, the authors believe that further stringent and well-designed investigations are required to respond to these observations, and identify the mechanism of the host–tumour responses observed during the early phase of the therapy, as well as to establish standardised laser and treatment protocols, with a safe laser dose and time factor, and long-term follow-up. 

It’s worth noting that in recent decades, PBMT has been utilised in OM management in a head and neck cancer (HNC) cohort without any reported adverse effects [37,38]. Nevertheless, the biological impact of PBM on tumour response and/or tumour behaviour, as well as the microenvironment of tumour cells, remains uncertain. Notably, the above in vitro cell culture studies revealed some contradictions in utilising PBMT, which are possibly related to the tumour’s genomic heterogeneity, when the light phonic energy is distributed uniformly [39]. However, further studies are required to provide a reliable explanation.

## 3. What Are the Significant Benefits of PBMT for H&N Oncology Patients?

### OM Induced by Cancer Therapies (RT-CT)

OM is a common adverse response to CT and/or RT, causing pain, difficulty in swallowing and eating, oral ulceration, and ultimately interruption of the course of treatments. It has shown that the etiopathology of mucositis is complex and multifactorial. In this context, there is a lack of in-depth understanding of the mechanisms of the contributing factors to mucosal disintegration [40]. The clinical management of OM is fundamentally palliative. However, the Multinational Association of Supportive Care in Cancer: MASCC/ISOO Mucositis Clinical Practice Guidelines, 2019, recommended several preventive and treatment approaches, as follows: Bland rinses (0.9% saline/sodium bicarbonates solution), Topical anesthesia (0.5% or 1.0% dyclonin hydrochloride), mucosal coating agents (Amphojel, Zilactin) and Analgesics (Opioid drugs, Benzydamine HCL topical rinse) [41]. Nevertheless, PBMT has been recommended for OM management for several reasons. There is consistent evidence, from a small number of high-quality studies, that suggest that red and infrared (IR) PBMT can partly prevent the development of cancer therapy-induced OM, and notably, can reduce pain intensity and promote healing [42]. The ultimate goal is to find measures that can block the development of pain or modulate the pain pathways. This perspective highlights the current recommended doses of various suitable wavelengths in utilising PBMT in the management of oral cancer therapy. 

Notwithstanding extensive evidence of the effectiveness of PBMT, it is still seeking global recognition and acceptance as a therapy. In this context, it is reliant on many influencing, factors such as various light sources, rigorous study design, and various laser parameter protocols, employed for each specific clinical application (irradiance, fluence, therapeutic power output, frequency and exposure time) [2]. The impact of these factors has led to a lack of consensus in establishing the standardised protocols. Thus, researchers are investing in trying to understand the phenomena of the laser light interactions with biological tissues, as well as observing the tissue responses to various wavelengths, in order to achieve the optimal therapeutic outcome. 

#### Data Extracted from Clinical Studies

Several clinical studies in the systematic review of Oberoi et al., 2014 [43], highlighted the efficacy of the photonic energy of the light source in modulating the oxidative stress, which is essential in determining the onset and progression of OM lesions. The clinical study of Ottaviani et al., 2013 [37], noted a great improvement in the clinical presentation of the OM lesions from the fifth day of treatment, when they irradiated it with a PBM 970-nm laser light. Moreover, ROS levels in the saliva of these patients revealed noticeable antioxidant activity induced in each PBM session. Nonetheless, the reduction in the ROS level was momentary but inconsistent during the first 24 h post-treatment. The results of PBMT in OM management are not entirely understood, and further research is needed.

The review of Sonis et al., 2014, reported on the role of PBMT in altering tumour growth and the spread of the malignancy, cellular level destruction, and the time taken to promote tissue healing. Based on the outcomes of its selected studies, the authors were not in favour of utilising PBMT for the management of OM, due to the influence of genetic pathways and the limited information on the safe use of PBMT [14]. Controversially, several PBMT treatment protocols have surfaced, emphasising the importance of laser dosimetry in achieving beneficial results for OM management. Although there is a large degree of variation amongst the protocols, the potential benefits of the frequently-utilised red laser wavelengths in treating OM cannot be overlooked [43]. 

The study of Oton-Leite et al., 2012 [44], utilised a 660-nm laser light, at 25 mW power output with 0.24 J of energy per point, daily until the end of the HNC therapy protocol, with satisfactory results. Moreover, PBMT in OM has been associated with lowering the frequency of oncological therapy interruption, shortening the recovery time, reducing the severity of OM [45] and enhancing healing [46]. Ultimately, this has led to a crucial benefit in improving a patient’s prognosis [47] and their QoL [25,48]. The long-term safety of low-level laser therapy, and its potential impact on tumour response, are still subject to testing.

The randomized controlled trails (RCT) studies of systematic review and meta-analysis in 2011 [42] highlighted that there is reliable evidence, from a small number of high-quality studies, that PBM with red and IR can play a partial role in preventing the eruption of OM induction by CT-RT. On this note, several studies have employed PBMT, with red or IR diode lasers at a power output between 10 and 100 mW, in the spotting technique, as preventive and therapeutic strategies. These results have shown the superiority of prophylactic (preventive) PBM application over the therapeutic approach [49,50]. This was confirmed by the MASCC/ISOO-recommended guidelines regarding OM induced by high-dose CT for hematopoietic stem cell transplantation (HSCT), and H&N RT without CT [25]. Moreover, further studies investigated the effectiveness of PBMT in the treatment of OM induced by H&N and hematological cancers, or mixed anti-cancer treatment modalities [51,52,53]. However, it is not currently possible to propose a consensus on standardised PBMT protocol, due to the substantial heterogeneity in the available data. 

A narrative review by Zecha et al., 2016 [54], evaluated the mechanism of action of PBM, dosimetry and safety considerations near the tumour site. The results were inconclusive for various reasons, including that the impact of PBM on tumour behaviour and response, and conflicting in vitro results regarding the effects of PBM on tumour cells. Ultimately, this could be attributed to inconsistencies in the PBM dosimetry and study designs, and methods of investigation. Nonetheless, the biological sources for broad clinical outcomes credited to PBM have also been identified as being similar to those activities and pathways associated with negative tumour behaviour, and hindered response to treatment [54].

A recent systematic review by Zadik et al., 2019 [55], established a PBM recommendation protocol for OM prevention in patients undergoing one of the following treatments: HSCT, H&N RT and H&N RT-CT. Two clinically effective PBMT protocols emerged; one for the red light (633–685 nm) and the other one for the IR (780–830 nm) (Table 2). Nonetheless, there is a lack of standardised PBMT protocol for intra-oral OM prevention in patients undergoing CT, as a result of a lack of evidence of RCTs and fundamental inconsistencies in the level of protein tyrosine phosphatase in reports of low-level clinical evidence-based practice [37,56,57,58].

Several studies evaluated PBMT as an alternative non-drug therapy for ORM management [21,59]. The primary human studies showed PBMT, in ORM induced by HNSCC, improved wound healing and pain alleviation, and ultimately the QoL [20,22,60]. It is important to note that there are no recommended PBMT guidelines for HNSCC patients undergoing RT, due to a lack of clarity concerning the impact of PBMT on the molecular structures of many cells. Moreover, very rare studies evaluated PBMT’s effects on dysphagia, oral dryness and taste alteration, as a result of cancer treatment [61]. Regarding this, a recent case series by El Mobadder et al., 2019 [62] utilised a PBMT protocol explained in Table 3, which has been shown to be effective in reducing dysphagia. 

## 4. Benefits of PBMT in Potentially Malignant Oral Lesions Management 

The management of oral premalignant or potentially malignant pathologies, such as oral leukoplakia and erythroplakia, as well as oral lichen planus (OLP), especially in the erosive form, remains a challenge in the long-term [63]. Their treatment might include one, or combined modalities, of the following: clinical observation, topical and systemic corticosteroids therapies, surgical excision, and laser ablation. The latter is considered the gold standard in treating clinically high-grade premalignant lesions [63,64]. It is important to note that there are less post-operative complications reported in laser treatment compared to the surgical excision [65]. One of these advantages is minimal post-operative pain, as the photonic energy of the laser light targets the vasculature of oral lesions, leaving mucosa intact. Several studies reported that various wavelengths, such as carbon dioxide ((CO_2_) 10,600 nm), 940-nm diode and 532-nm pulsed potassium-titanyl phosphate (KTP), are utilised to treat clinically selected oral premalignant lesions [66,67]. On this note, the CO_2_ laser, as an ablative tool, has been utilised to treat oral leukoplakia with mild dysplastic tissue, with no adverse effects. In a prospective study, Jerjes et al., 2012 [67], assessed the use of laser surgery with CO_2_ in treating oral premalignant lesions, in terms of their recurrence rate, residual tumour transformation, and the overall clinical outcomes based on more than 6 years of follow-ups. The results identified that erythroplakias and non-homogenous leukoplakias mainly show a degree of recurrence and malignant transformation. Nonetheless, laser resection/ablation modalities for oral dysplasia are recommended, not only to prevent recurrence and malignant transformation, but also to minimise post-operative oral dysfunction, which can be encountered using the conventional modalities [67]. Currently, there is limited evidence to suggest that PBMT can be utilised as an oral leukoplakia treatment modality. The study of Arnaoutakis et al., 2013, reported that surgical excision of OLP lesions has an advantage in preventing the mutation of the cells, compared to other treatment modalities [68]. 

The recent systematic review by Garcia-Pola et al., 2017 [69], evaluated the therapeutic efficacy of PBMT on alleviated pain, and the clinical signs of erosive and atrophic forms of OLP. The outcome of this systematic review demonstrated a therapeutic framework for OLP, which is as follows: the first line of treatment is clobetasol propionate at 0.025–0.05% for topical use; secondly, 0.1% tacrolimus and 1% pimecrolimus is used in the topical format; and finally comes the systemic corticosteroids, and the application of diode lasers therapy [69]. Several studies have achieved positive effects in reducing the main symptoms of OLP, in terms of pain, lesion size and redness, but unfortunately none have achieved the resolution of the lesion and a cure [70,71]. This was supported by a systematic review by Al-Maweri et al., 2017, which stated that PBMT can assist in reducing the symptoms associated with OLP when performed with wavelengths ranging from 630 to 980 nm, employed at a power output from 20 to 300 mW, and with exposure time ranging from 10 s to 15 min [72]. 

There is conflicting data in the literature regarding PBMT’s effectiveness and topical corticosteroids in OLP management. The study of Cafaro et al., 2010, reported that PBMT is effective in reducing symptoms OLP lesions, which are unresponsive to topical corticosteroids [71]. Nevertheless, a recent systematic review by Akram et al., 2018, aimed to evaluate the effectiveness of PBMT compared to topical corticosteroids in atrophic-erosive forms of OLP management [73]. All nine of the studies included in the review reported the following PBMT protocol: wavelengths between 630 nm and 970 nm, power output of 10–3000 mW, range of spot sizes of 0.2–1.0 cm^2^, and 6 –480 s exposure time. These were effective in the treatment of OLP patients, with a follow-up period ranging from 4 to 48 weeks. Three of these studies showed significantly higher improvements in the clinical presentation with topical use of corticosteroids, compared to PBMT, while one study showed significant improvement with PBMT. One study showed comparable outcomes between PBMT and corticosteroid. Controversially, Kazancioglu et al., 2015, conducted an RCT study (100 subjects recruited) [74] comparing ozone (60% for 10 s for 10 sessions) to PBMT (λ 808 nm, 0.1 W, CW, 120 J/cm^2^, 150 s, at a distance of 0.5 cm, and 10 sessions treatment duration) in the management of atrophic-erosive OLP (≤3 cm). The positive control group was treated first of all with a corticosteroid mouthwash (dexamethasone) for five minutes, and 30 min later with a mouth rinse with nystatin solution (100,000 units mycostatin oral suspension, four times a day for one month), while the negative control were treated with a solution free from any medications (rinsing for five minutes, four times a day for one month). The follow-up was up after a six-month period. The results showed an improvement of the symptoms (pain and redness) for PBM, ozone and corticosteroids; however, statistical significance in symptom improvement was only noted in the ozone and corticosteroids groups. Therefore, it remains debatable whether PBMT is more effective, compared to corticosteroids, in the management of OLP (atrophic-erosive forms), considering that the current scientific evidence is weak. 

It is important to note that PBMT is not a “cure” for OLP or oral leukoplakia lesions. In the absence of significant data and long-term follow-up clinical studies, it provides benefits in enhancing QoL. After extensive research into the literature, and to the best of the knowledge of the authors, no evidence-based practice or medicine suggests that PBMT can potentially cure the premalignant lesion, due to the heterogeneity of the available evidence-based literature and the lack of long-term follow-ups. More research is required in order approve it for clinical use. Nevertheless, PBM appears to be a promising modality in minimising the clinical symptoms of premalignant oral lesions.

## 5. Conclusions and Future Perspectives

The authors conclude that the long-term safety of PBM therapy, and its potential impact on H&N tumour response, is still under consideration. The authors’ recommendations for future studies are to have a better understanding of the cellular and molecular mechanisms and light behaviour, as well as to evaluate and ensure the safety of this therapy near the tumour site in the H&N region. This review highlighted that there is growing evidence for the clinical benefits of PBMT in enhancing the QoL of oncology patients. Therefore, PBMT is a promising treatment modality, which needs to be further validated (due to the heterogeneity in the available data and a lack of standardised PBM protocol) by well-designed and long-term RCTs, which are evaluated by their diligent and impartial outcomes, and ensure laser irradiation safety at the tumour site.

## Figures and Tables

**Table 1:** The critical issues that require consideration in photobiomodulation therapy (PBMT) [9].

Biological			Clinical	
Technical	Molecular	Cellular/tissue	Device	Delivery
Scale Kinetics Background	Target Regulation	Context	Wavelength Polarisation Coherence Fluence Irradiance Time Pulsing	Clinical treatment site Delivery method (fixed/moving) Depth of target Dose repetition Biomarkers Off-target (bystander) effects

**Table 2:** Summarised recommended treatment parameters. Abbreviations: nm (nanometer), mW (milliwatt), J (joule), cm^2^ (centimeter square), J (Joule), s (second).

Wavelength (nm)	Power Output (mW)	Spot Size (cm^2^)	Energy per Point (J)	Maximum Irradiation per Point (s)	Maximum Number of Irradiation Points	Minimal Sessions per Week During Cancer Treatment	Maximum Number of Days to Start PBMT, before Cancer Therapy (for Prevention)
Red (633–685)	10–60	0.1–1.00	3	30	6	3	7
Infrared (780–830)	50–11	0.1–0.5	6	30	6	3	7

**Table 3:** Applications and treatment protocols (intra- and extra-oral approaches) of PBMT in dysphagia treatment.

Irradiation Site	Trigger Points	PBMT Protocol
Intra-oral	Bilaterally, four points to soft palate and onto oropharynx.	635 nm, 3 J/cm^2^, 10 s exposure time on each point, 100 mW, continuous and contact mode.
Extra-oral	Lateral and ventral surfaces of the pharynx and larynx. Midline and lateral aspects of neck	
